# Evaluating Hand Function in Clients with Trigger Finger

**DOI:** 10.1155/2017/9539206

**Published:** 2017-01-10

**Authors:** Danit Langer, Adina Maeir, Michael Michailevich, Shai Luria

**Affiliations:** ^1^School of Occupational Therapy, Hadassah and Hebrew University, Jerusalem, Israel; ^2^Department of Orthopaedics, Sherutay Briut Clalit, Jerusalem, Israel; ^3^Department of Orthopedic Surgery, Hadassah-Hebrew University Medical Center, Jerusalem, Israel

## Abstract

**Background:**

Trigger finger (TF) is a common hand pathology frequently encountered in hand clinics. Occupational therapists predominantly assess TF symptoms as opposed to using standardized hand functioning assessments. The purpose of this study was to assess the construct validity of dexterity and grip strength assessments for clients with TF.

**Method:**

Sixty-three participants with TF and 66 healthy controls were administered the Functional Dexterity Test (FDT), Purdue Pegboard Test (PPT), and Jamar® Hydraulic Hand Dynamometer (JD) and completed the Disabilities of Arm Shoulder and Hand questionnaire (DASH). TF symptoms were graded using the Quinnell classification.

**Results:**

Statistically significant differences were found between the groups in dexterity and grip strength. A statistically significant difference between the three TF grades was found on the PPT. All three test scores were moderately correlated with the DASH scores.

**Conclusion:**

This study provides innovative evidence for the validity of common hand function assessments for individuals with TF and recommends incorporating these tools in clinical practice. Further research is needed with larger samples and better representation of each TF clinical grade.

## 1. Introduction

Hand function is a broad term that incorporates several components, including strength, sensation, range of motion, and dexterity. Normal function of the hand is an important factor in a person's ability to independently engage in daily activities and occupations [1]. Damage to one or more of these components can lead to dysfunction of the hand and limit participation in everyday life [2]. According to the International Classification of Function (ICF), there is an interaction between the elements of* body systems* and* structures* (such as dexterity and strength),* activity* and* participation* in life roles, and* contextual factors* (environmental factors and personal factors) [3].

Trigger Finger (TF), also termed Stenosing Flexor Tenosynovitis, is one of the most common pathologies seen in hand surgery clinics and is the fourth leading cause of referral to these clinics [4]. Triggering of the finger commonly occurs at the fibroosseous tunnel formed by the metacarpal neck and the first annular pulley. The initial complaints associated with TF are pain over the A1 pulley or clicking and may worsen to severe pain and locking of the digit in flexion [5]. The incidence of TF is 28 : 100,000 per year or a lifetime risk of 2.6% in the general population, but it increases to 10% in the diabetic population [6]. The mean age of onset for TF is 58 years and it is diagnosed in women two to six times more frequently than men [5]. The diagnosis of TF is based on symptoms and physical examination, yet in recently published consensus guidelines for managing TF no uniform grading system has been recommended [7]. There are a variety of methods to treat TF; interventions include both nonsurgical and surgical treatment options [8]. In recently published guidelines, experts agreed on the following methods for the management of TF: orthotics, corticosteroid injections, and surgical treatment. When planning a treatment regimen, they recommend considering the severity and duration of the pathology as well as previous treatment [7].

The majority of the intervention studies regarding TF utilized symptom resolution as the outcome measure, as opposed to utilizing hand function outcomes, such as dexterity and strength [9–12]. A similar trend was found among a cohort of 61 occupational therapists from Israel and the United States, who reported on their assessment practices for clients with TF. These therapists predominantly evaluate TF symptoms, as opposed to the use of standardized hand functioning assessments in clients with other hand conditions [13]. This trend could be attributed to the fact that assessments for hand function have not been validated for individuals with TF. In addition, the lack of validated measures may impede documenting and establishing evidence based occupational therapy for treating individuals with TF [14]. Therefore, the aim of the present study was to assess the construct validity of tools for measuring several components of hand function in clients with TF.

The focus of this study is on hand function measures of dexterity and strength. Dexterity is the ability to use your hands skillfully and is defined as fine, voluntary movement used to manipulate small objects during a specific task [15]. Dexterity can be subcategorized into gross manual dexterity, which is the ability to handle objects with the hand and into fine motor dexterity, which refers to in-hand manipulations using the thumb and second or third digit (McPhee in [16, 17]). Grip strength is defined as the force applied by the hand and fingers or the measurable ability to exert pressure on objects. In the consensus guidelines for the assessment of clients with hand conditions it was agreed that the Jamar Hydraulic Hand Dynamometer (JD) and the Pinch Gauge device should be used to assess strength, yet no consensus was reached regarding the assessment of dexterity [18]. The evaluation of dexterity can provide information about the neuromotor function of the hand, since sensation and intrinsic hand strength are essential for performing manipulative movements [15].

There are many methods available to assess dexterity and the selection of an appropriate tool is often based on a variety of factors, including availability, familiarity, and applicability to a given population and psychometric soundness. For the assessment of dexterity in the present study we used the Functional Dexterity Test (FDT) and the Purdue Pegboard Test (PPT) whose psychometric properties have been well established in other targeted health populations [1, 15] and have been reported to be commonly used by occupational therapists in hand clinics [13].

*Study Objectives*. The objectives of the study were to assess the construct validity of the FDT, PPT, and the JD for people with TF, specifically to (a) evaluate the ability of these measures to distinguish amongst grades of TF severity; (b) to distinguish between groups with and without TF; (c) to assess whether the side of the involved hand (radial or ulnar) effects grip strength and dexterity; and (d) finally to evaluate the correlation between measures of hand function and self-reported disability. Accordingly the research questions were as follows: (a) Will statistically significant differences be found between the three TF grades in the scores of the FDT, PPT, and JD? (b) Will statistically significant differences be found between the TF and control groups in the scores of the FDT, PPT, and JD? (c) Will statistically significant differences be found between participants with TF on the radial side and the ulnar side in the scores of the FDT, PPT, and JD? (d) Will statistically significant correlation be found between the DASH score and the FDT, PPT, and JD scores of the TF group?

## 2. Method

### 2.1. Study Design

The study was a cross-sectional study.

### 2.2. Participants

The study protocol was approved by the Institutional Helsinki Committee. One hundred and fifty consecutive clients with TF that presented to a central orthopedic clinic of a major health maintenance organization (HMO) and an outpatient clinic in a major hospital between March 1st 2012 and April 30th 2013 were invited to participate in the study. Inclusion criteria were adult clients (age 18 years or above) with a diagnosis of one or more digits with TF of a Quinnell grade one or higher. According to the Quinnell grading system, TF fingers are rated as follows: 0, normal movement of the digit; 1, uneven movement; 2, actively correctable locking of the digit; 3, passively correctable locking; and 4, fixed deformity [19]. The exclusion criteria were upper extremity trauma in the preceding year, known neurological deficits, known cognitive deficits, and pregnant women. Written informed consent was obtained from all participants in the study. The demographic and clinical data of the participants were recorded. Participants were classified as having additional medical conditions if they reported one or more medical condition besides TF. The severity of the TF was recorded by the hand surgeon using the Quinnell grading system. Consenting TF participants were administered the FDT, PPT, JD, numeric pain scale, and the Disabilities of the Arm Shoulder and Hand (DASH) questionnaire immediately after their visit with the doctor. A control group of 66 healthy participants was recruited using a convenience sample and matched for age and gender to the research group. The TF and control groups data was previously analyzed and published [20, 21]. The results of these two studies demonstrated (a) the effect of TF severity on functioning and quality of life as measured by the DASH and the World Health Organization Quality of Life Brief questionnaire (WHOQ-BRIEF) [21] and (b) the wide impact of TF on hand functioning, activities of daily living, and quality of life [20].

### 2.3. Measures

#### 2.3.1. Functional Dexterity Test (FDT)

The FDT tool is suitable for the adult population, 20–70 years old, with various injuries to the upper limb. The FDT gives information regarding the clients' ability to use their hands for functional tasks requiring a dynamic 3-jaw chuck grasp pattern. It is made of a square wooden pegboard with 16 pegs. The examiner documents the time required to turn over all the pegs. Execution time is measured on each hand separately. A five-second penalty is added every time the participant supinates or touches the board and a 10-second penalty is added if the participant drops a peg. Two scores are obtained for each hand: the net time in seconds and the total score (net time plus penalties). The net time score was used in the present study. The FDT was found to have good interrater and good test retest reliability [16, 22].

#### 2.3.2. Purdue Pegboard Test (PPT)

The PPT was developed in 1948, in order to assess manual dexterity and precision of applicants for industrial work [23]. Since then, the PPT has been used in rehabilitation and in research [24]. The PPT includes four subtests; in the first three subtests participants are asked within 30 seconds to place the maximum number of metal pins, one at a time, into a row of pegboard holes, first with their dominant hand and then with their nondominant hand followed by placing pairs of pins with both hands simultaneously. In the fourth and final subtest, the participant uses alternate hands to form the maximum number of assemblies consisting of a pin, washer, collar, and second washer within 60 seconds. The PPT tests the quality and speed of performance of the hand as the person accomplishes the four subtests [23].

#### 2.3.3. Jamar Hydraulic Hand Dynamometer (JD)

The JD was designed to measure gross power fist grip and is considered to be the most accurate test for this skill. The American Society of Hand Therapists (ASHT) recommended the use of this tool for the assessment of grip strength [25]. The test was administered with the guidelines of the ASHT. Psychometric testing found good interrater reliability and high test retest reliability [26].

#### 2.3.4. Disabilities of the Arm Shoulder and Hand (DASH)

The DASH was developed in order to describe the disability experienced by people with upper-limb disorders and also to monitor changes in symptoms and function over time. The questionnaire consists of 30 questions related to physical function, social function, and different symptoms. Each item has five response options. The scores for all items are then used to calculate a scale score ranging from 0 (no disability) to 100 (most severe disability). There are two additional parts with four questions that are relevant for people that engage in sports, music, and work [27]. These parts were not included in the present study.

#### 2.3.5. The Quinnell Grading System

The Quinnell grading system is used to assess clinical severity of TF. According to this classification, TF fingers are rated as follows: 0, normal movement of the digit; 1, uneven movement; 2, actively correctable locking of the digit; 3, passively correctible locking; and 4, fixed deformity [19].

#### 2.3.6. Numerical Pain Rating Scale (NRS)

The NRS is a scale with 11 degrees, reflecting the subjective intensity of pain experienced by a person during the preceding day or the previous week (see Figure 2). The pain scale can be administered verbally or by using a visual scale [28].

### 2.4. Data Analysis

The distribution of variables in the study (FDT, PPT, JD, pain, and age) met the criteria for normality based on the Shapiro-Wilk test of normality (*p* > 0.05). Prior to main hypotheses testing, between groups comparisons of demographics and background clinical data were performed in order to rule out extraneous factors (demographics and background clinical data) that might influence the results. Analysis of variance (ANOVA) was used to compare age and pain intensity between the TF subgroups (TF grades 1–3). A Chi-Square analysis was used to compare gender, hand dominance, affected hand, and presence of additional medical conditions between study and control group and between TF subgroups.

For the hypotheses testing, one-way ANOVA was used in order to compare the mean scores (FDT, PPT, and JD) obtained by the different TF grades and post hoc Tukey HSD test comparisons between groups were conducted. The TF group was divided according to affected finger. If the affected finger was the thumb index or middle finger the participant was assigned to the radial side affected group and if affected finger was the ring or small finger the participant was assigned to the ulnar side affected group. An independent sample* t*-test was used to compare FDT, PPT, and JD between radial and ulnar affected side groups. The correlations between the FDT, PPT, JD, and DASH were calculated using Pearson's correlations. Effects sizes were calculated according to the statistical test, partial eta squared for ANOVA [29], and Cohen's d for* t*-test and correlation [30].

### 2.5. Procedure

Informed consent was obtained by hand surgeons. The hand functioning assessments and questionnaires were administered by experienced occupational therapists to participants immediately after their visit with the doctor. The occupational therapists were trained in the administration of the assessments and were tested for correct administration by the head researcher. Healthy participants were administered the same assessment protocol.

## 3. Results

**Figure 1:**
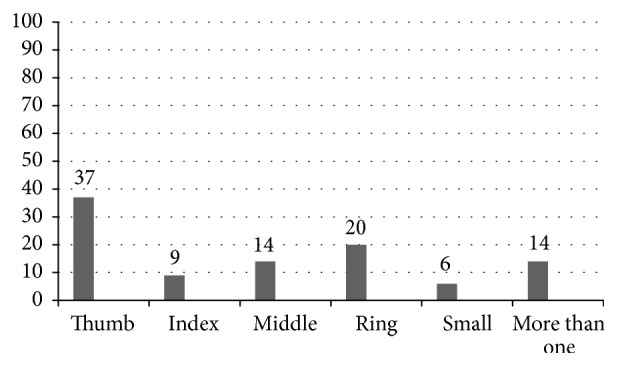
Distribution of Trigger finger digits.

**Figure 2:**
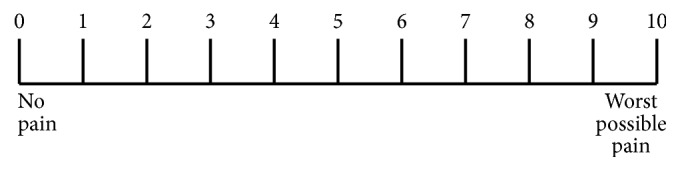
Numerical Pain Rating Scale.

Of 150 clients presenting with TF during the study period, a total of 63 met the inclusion criteria, agreed to participate in the study, and completed all assessments. The study group included participants with TF grades 1–3. Demographic and clinical data of participants are presented in Table 1 and distribution of affected digits in Figure 1. No statistically significant differences in demographic and background clinical data were found between subgroups of TF grades, between healthy and control groups, and between radial affected side and ulnar affected side groups (*p* > 0.05).

### 3.1. Differences in Hand Function between TF Grades

A statistically significant large effect of TF grade was found for PPT subtests demonstrating a decrease in PPT scores with increasing severity of TF (*p* < 0.05) (see Table 2). Post hoc comparisons revealed statistically significant differences between grades 1 and 3 TF subgroups on the affected hand, both hands, and assembly subtests. For both hands subtest a statistically significant difference was also found between grades 2 and 3. As for the FDT, a nonstatistically significant trend was found of increased scores (a higher score reflects reduced dexterity) with higher grade of pathology severity. As for grip strength no group effect was found (see Table 2).

### 3.2. Decreased Hand Function in TF Group versus Control Group

A statistically significant difference was found between the TF group and controls in all tests scores. The control group achieved superior scores in all the tests (see Table 3).

### 3.3. Differences in Hand Function between TF Participants with Radial versus Ulnar Side Affected

There were 44 participants with TF on the radial side of the hand and 19 with TF on the ulnar side. No statistically significant differences were found between these two groups in all the measures (see Table 4).

### 3.4. Dexterity and Grip Strength Test Scores Correlate with Disability Score

Statistically significant moderate correlations were found between the DASH and all the test scores of the affected hand in the TF group except for the PPT affected hand subtest (see Table 5).

## 4. Discussion

The purpose of the study was to evaluate the construct validity of the FDT, PPT, and JD for participants with TF. The results of the present study demonstrated that all three tools discriminated between people with and without TF. However, only the PPT had a statistically significant group effect on the clinical grades. Statistically significant differences were found between clinical grades 1 and 3 for all subtests of the PPT. No statistically significant differences were found between participants who had TF on the radial or ulnar side. Furthermore, all the tools (excluding one PPT subtest) were statistically significantly correlated with the DASH.

The results of the present study demonstrated the impairment in dexterity among individuals with TF in comparison to controls and that this impairment increased with the severity of TF. The FDT and PPT are both commonly used in clinical settings [13] yet there is a paucity of studies that examined the degree to which these tools are valid for assessing populations with specific hand conditions [24]. The current findings support the validity of the PPT in the TF population and are in line with studies of dexterity in individuals with other hand conditions. A study that examined the validity and reliability of the PPT for people with Carpal Tunnel Syndrome (CTS) found an association between disease severity and all the subtests of the PPT in patients aged 60 and older and statistically significant differences between the CTS group and controls [31]. An earlier study conducted by Ben Shahar et al., in 1998, also found that the PPT differentiated between a group of clients with a variety of hand conditions and controls. In addition to the evidence obtained regarding construct validity, the findings of this study also support the ecological validity of the PPT for individuals with TF as demonstrated by moderate correlations between PPT subtests and the DASH. These findings are in line with previous studies that demonstrated moderate correlations between the PPT and Activities of Daily Living (ADL) [24, 32]. However, a recent study by Gonzalez et al. [33] demonstrated that the grasping patterns of the fingers while preforming the PPT differed from the patterns observed while performing ADL. The authors suggest that traditional hand function assessments may not capture the complex hand performance that is essential for completing ADL. These findings are in line with the results of the present study that demonstrated only a moderate correlation between the PPT and the DASH. These findings stress the importance of conducting a broad assessment that includes all parts of the ICF model when treating clients with hand conditions.

The findings regarding grip strength differed from dexterity, as no group effect of the clinical grades was demonstrated on the JD. However, TF participants had statistically significant weaker grip strength than the control group. Furthermore, a moderate statistically significant correlation was found between the JD and the DASH, supporting its ecological validity for the TF population. We were unable to find similar studies which used the JD with TF participants. However, several studies have demonstrated a similar trend of decreased grip strength among people with upper extremity musculoskeletal disorders in comparison to healthy controls [34]. Regarding correlation between grip strength and ADL, similar correlations were demonstrated in studies of elderly and individuals after distal radial fracture [35, 36].

## 5. Conclusions

The current findings suggest that the PPT and FDT are valid tools for measuring dexterity and the JD is a valid measure of strength in clients with TF. According to the results of the current study the PPT was more sensitive to the clinical grades of TF than the other measures. The PPT, FDT, and JD were moderately correlated with a measure of disability (DASH). These findings are important in light of recent findings whereby a very low percentage of occupational therapists use these assessments in their practice with clients with TF, despite the fact that they commonly use these tools with other clients [13]. Based on the findings of the present study therapists may consider incorporating these measures into their assessment protocol for clients with TF.

## Figures and Tables

**Table 1:** Demographic and clinical data: comparison of TF subgroups.

	TF (*n* = 63)	TF 1 (*n* = 11)	TF 2 (*n* = 43)	TF 3 (*n* = 9)	Control (*n* = 66)
	Mean (SD)	Mean (SD)	Mean (SD)	Mean (SD)	Mean (SD)

Age	60.48 (11.34)	57.08 (16.8)	60.53 (9.87)	63.44 (10.87)	58.60 (11.55)
Pain	5.52 (2.45)	5.82 (1.53)	5.48 (2.78)	5.33 (1.58)	

	*n* (%)	*n* (%)	*n* (%)	*n* (%)	*n* (%)

Gender					
Male	20 (32)	5 (45)	13 (30)	2 (22)	23 (35)
Female	43 (68)	6 (55)	30 (70)	7 (78)	43 (65)
Dominance					
Right	56 (89)	10 (91)	40 (93)	6 (67)	57 (86)
Left	7 (11)	1 (9)	3 (7)	3 (33)	9 (14)
Affected Hand					
Dominant	36 (57)	9 (82)	21 (49)	6 (67)	
Nondominant	17 (27)	1 (9)	14 (33)	2 (22)	
Both	10 (16)	1 (9)	8 (18)	1 (11)	
Additional conditions					
Yes	34 (54)	5 (45)	24 (56)	4 (44)	27 (41)
No	29 (46)	6 (55)	19 (44)	5 (56)	39 (59)

TF = trigger finger.

**Table 2:** Mean difference in FDT, PPT, and JD scores amongst different groups TF grades.

	TF grades											
	TF 1 (*n* = 11)		TF 2 (*n* = 42)		TF 3 (*n* = 9)		ANOVA			I versus II	I versus III	II versus III
	Mean (SD)	95% CI	Mean (SD)	95% CI	Mean (SD)	95% CI	*F*	*p*	*η*_*p*2_	*p*	*p*	*p*
FDT affected hand	28.34 (4.98)	13.22–43.45	37.41 (28.74)	29.67–45.14	47.39 (21.43)	30.68–64.11	1.453	0.246	0.046	0.568	0.247	0.558
PPT affected hand	13.18 (2.04)	11.83–14.53	11.55 (2.13)	10.86–12.24	10 (2.87)	10.86–12.24	5.083	0.009^*∗*^	0.147	0.106	0.010^*∗*^	0.177
PPT both hands	10.64 (1.21)	9.55–11.73	9.38 (1.89)	8.82–9.94	7.67 (1.94)	8.82–9.94	6.705	0.002^*∗*^	0.185	0.131	0.002^*∗*^	0.042^*∗*^
PPT assembly	28.36 (7.55)	25.12–31.61	23.88 (4.68)	22.22–25.54	19.22 (5.4)	15.64–22.81	7.191	0.002^*∗*^	0.196	0.056	0.002^*∗∗*^	0.070
JD affected hand	21.4 (5.29)	15.72–27.01	16.25 (9.87)	13.34–19.16	16.61 (9.48)	10.33–22.9	1.324	0.274	0.043	0.280	0.531	0.995

TF = trigger finger; FDT = Functional Dexterity Test; PPT = Purdue Pegboard Test; JD = Jamar Hydraulic Hand Dynamometer.

^*∗*^ *p* ≤ 0.05; ^*∗∗*^*p* ≤ 0.001.

**Table 3:** Mean difference in in FDT, PPT, and JD among TF and control groups.

	TF group mean (SD)	Control group mean (SD)	*t*_(df)_	*p*	Effect size	95% CI of the difference
FDT DH (*n* = 44)	36.5 (28.3)	27.7 (8.5)	2.0_(108)_	0.05	0.421	16.1–1.4
FDT NDH (*n* = 19)	38.8 (15.8)	29.2 (7.7)	2.6_(83)_	0.017	0.772	17.5–1.9
PPT DH (*n* = 44)	11.9 (2.5)	13.5 (2.5)	3.3_(108)_	0.001	0.639	0.6–2.5
PPT NDH (*n* = 19)	10.6 (1.9)	13.0 (2.4)	4.1_(83)_	0.001	1.108	1.3–3.6
PPT BH (*n* = 63)	9.3 (2)	10.5 (2.1)	3.3_(127)_	0.002	0.585	0.5–1.9
PPT assembly (*n* = 62)	24 (5.9)	27.5 (8.23)	2.8_(126)_	0.006	0.489	1.1–6.01
JD DH (*n* = 44)	19.2 (8.8)	28.5 (10.2)	4.9_(108)_	0.001	0.976	5.6–13.1
JD NDH (*n* = 19)	11.1 (8.5)	26.4 (10.1)	6.1_(83)_	0.001	1.639	10.3–20.4

TF = trigger finger; FDT = Functional Dexterity Test; PPT = Purdue Pegboard Test; JD = Jamar Hydraulic Hand Dynamometer; DH = dominant hand; NDH = nondominant Hand; BH = both hands.

**Table 4:** Mean difference in FDT, PPT, and JD scores between TF participants with radial versus ulnar side affected.

	Radial affected side group *n* = 44	Ulnar affected side group *n* = 19	*p*
	Mean (SD)	Mean (SD)	
FDT affected hand	40.7 (18.5)	45.5 (39.4)	NS
PPT affected hand	11.6 (2.7)	11.4 (1.7)	NS
PPT both hands	9.3 (2.1)	9.3 (1.8)	NS
PPT assembly	23.7 (6.1)	24.7 (5.3)	NS
JD affected hand	16.6 (8.6)	19.4 (11.6)	NS

FDT = Functional Dexterity Test; PPT = Purdue Pegboard Test; JD = Jamar Hydraulic Hand Dynamometer.

**Table 5:** Correlations between FDT, PPT, and JD scores and DASH scores in TF group.

	FDT affected hand	PPT affected hand	PPT both hands	PPT assembly	JD affected hand
DASH	0.301^*∗*^	−0.214	−0.418^*∗∗*^	−0.350^*∗∗*^	−0.472^*∗∗*^

^*∗*^ *p* < 0.05; ^*∗∗*^*p* < 0.01.

FDT = Functional Dexterity Test; PPT = Purdue Pegboard Test; DJ = Jamar Hydraulic Hand Dynamometer; DASH = Disabilities of the Arm Shoulder and Hand.

## Additional Points

*Study Limitations and Recommendations*. The distribution of participants among the TF grades was not equal. Therefore, the findings of this study should be interpreted with caution. Further studies with larger representation of TF grades are recommended.
